# A Case of a Second Intermetatarsal Space Gouty Tophus with a Presentation Similar to a Morton’s Neuroma

**DOI:** 10.7759/cureus.2620

**Published:** 2018-05-14

**Authors:** Fatemeh Razaghi, Eildar Abyar, Carly A Cignetti, Jeffery A Jones, Eva Lehtonen, John L Johnson, Matthew Anderson, Alan Hsu, Kyle D Paul, Ashish Shah

**Affiliations:** 1 Department of Orthopedic Surgery, University of Alabama at Birmingham, Birmingham, USA; 2 School of Medicine, University of Alabama at Birmingham, Birmingham, USA; 3 Department of Pathology, University of Alabama at Birmingham, Birmingham, USA; 4 Miller School of Medicine, University of Miami, Miami, USA; 5 Orthopaedic Surgery, University of Alabama at Birmingham, Birmingham, USA

**Keywords:** gout, tophus, tophi, intermetatarsal, foot, morton's neuroma, lesion, surgery

## Abstract

Non-infectious soft tissue lesions of the foot and ankle are relatively rare clinically. These include benign and malignant neoplasms, as well as non-neoplastic or pseudotumoral lesions such as ganglionic, synovial and epidermoid cysts, intermetatarsal and adventitious bursitis, inflammatory lesions like gouty tophi and rheumatoid nodules, Morton’s neuroma, and granuloma annulare.

A 48-year-old male with a history of medically treated tophaceous gout presented with left foot neuropathic pain and paresthesia, in the setting of a well-circumscribed soft tissue lesion of the second intermetatarsal space, suspected to be a Morton’s neuroma. Magnetic resonance imaging (MRI) showed a 4.1 x 2.7 x 2.6 cm heterogeneous soft tissue mass containing multiple cystic areas. Excisional biopsy was performed and histologic examination revealed well-circumscribed nodules of amorphous material containing needle-shaped clefts, rimmed by histiocytes, and multinucleated giant cells consistent with a gouty tophus.

This is the first case reported in the literature of an intermetatarsal gouty tophus causing neuropathic pain and paresthesia. While Morton’s neuroma is the most common cause of this presentation, this case illustrates that other pseudotumoral lesions, such as a gouty tophus, may present similarly, and should be considered in the differential diagnosis. While most cases of tophaceous gout can be adequately treated with urate-lowering therapy, surgery may be indicated for tophi that do not resolve with medical treatment based upon symptom severity, compression of nearby structures, and functional impairment.

## Introduction

Non-infectious soft tissue lesions of the foot and ankle are relatively rare clinically. These include benign and malignant neoplasms as well as non-neoplastic or pseudotumoural lesions. The pseudotumoural lesions of the foot include ganglionic, synovial and epidermoid cysts, intermetatarsal and adventitious bursitis, inflammatory lesions such as gouty tophi and rheumatoid nodules, Morton’s neuroma, and granuloma annulare.

Gout is a disease characterized by high blood uric acid levels and monosodium urate crystal deposits in joints and extra-articular tissues [1]. This chronic condition is often associated with the presence of gouty tophi, which are amorphous or crystalline masses of urate with a surrounding layer of inflammatory tissue [2]. The tophus represents an organized chronic foreign body granulomatous inflammatory response to monosodium urate crystals. Tophaceous gout is a pseudotumoural process that usually manifests in later stages of this disease.

Clinical history, laboratory examination, and radiographic findings can help lead the clinician to the correct diagnosis of tophaceous gout. However, a gouty tophus may be mistaken for another pseudotumoral lesion or an infectious process. Gouty lesions most often occur in the foot at the metatarsophalangeal (MTP) or interphalangeal joints [3]. However, this condition has been called "the great pretender", due to the wide spectrum of clinical presentations that have been described [4]. The purpose of this case report is to describe a case of a gouty tophus atypically located at second intermetatarsal web space, with a presentation similar to a Morton’s neuroma.

## Case presentation

A 48-year-old male presented with left foot pain which started “a couple of years ago” with no injury. The patient first noticed a “knot” on his foot between the second and third toes approximately one year ago. The patient was referred to the orthopaedics foot and ankle clinic for further evaluation. The patient reported his pain as neuropathic and paresthetic, and radiating to the second interdigital space. His symptoms were worsened by walking, standing, and climbing ladders and stairs. The patient had a known history of gout and was currently being treated with allopurinol and nonsteroidal anti-inflammatory drugs (NSAIDs). Despite treatment with urate-lowering medication, the patient exhibited tophaceous lesions of various sizes on his left elbow, right knee, right foot and bilateral hands, all asymptomatic.

Upon evaluation, the patient was afebrile; the other vital signs were within normal limits. On physical examination, the patient had pain with palpation of the interspace between the second and third metatarsal heads, with no metatarsal-phalangeal instability or hyperkeratosis. The rest of the physical examination was unremarkable.

**Figure 1:**
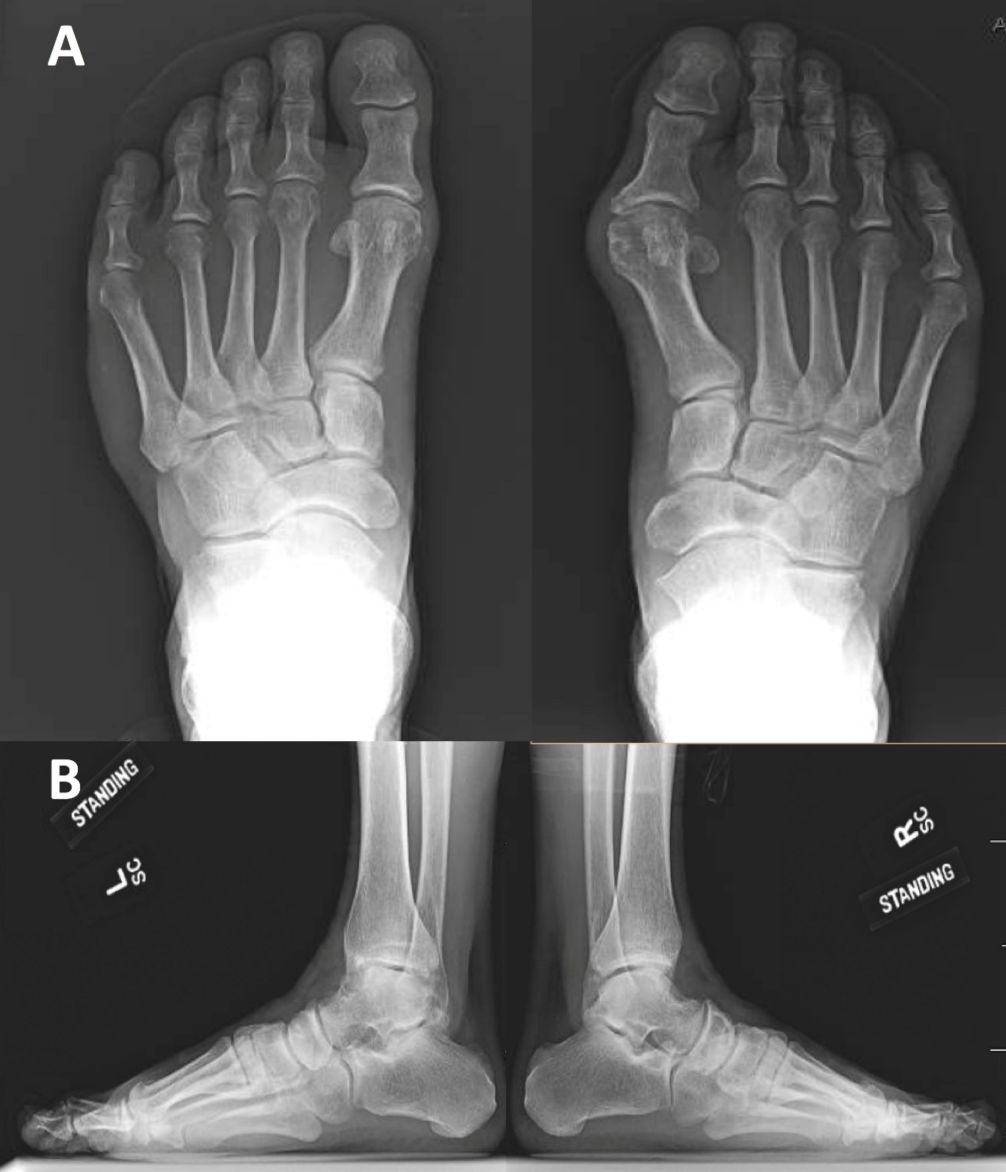
Pre-operative X-ray of bilateral feet, standing: (A) anterior-posterior and (B) lateral views

**Figure 2:**
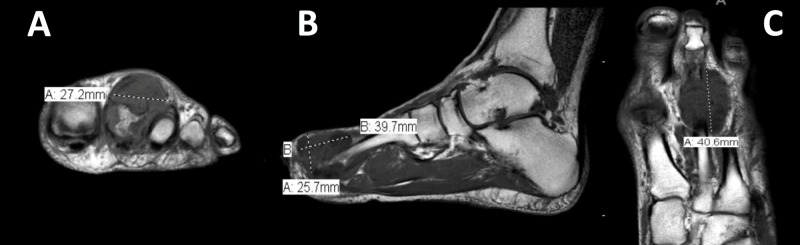
Pre-operative magnetic resonance imaging (MRI) of the left foot without contrast: (A) coronal, (B) sagittal, and (C) axial images at the level of the lesion with measurements

X-rays and magnetic resonance imaging (MRI) were ordered to supplement the physical examination. The anterior-posterior and lateral foot X-rays showed small periarticular erosions in the second metatarsophalangeal (MTP) joint, consistent with crystal-induced arthropathy, with no significant degenerative change, fracture, or dislocation (Figure 1). The MRI study showed a well-circumscribed, heterogeneous, soft tissue mass overlying the dorsal aspect of the second MTP joint, containing multiple internal cystic areas. The lesion was measured approximately 4.1 x 2.7 x 2.6 cm, based upon coronal, sagittal and axial T1 images (Figure 2). There was an extensive erosion of the second metatarsal head with associated cortical destruction. The patient’s serum uric acid level was 6.2 mg/dL (normal range 4.0-8.5 mg/dL).

The patient was consented for an excisional biopsy of the lesion and decompression of the second intermetatarsal space. The procedure started with a dorsal incision over the second intermetatarsal space. The skin was sharply dissected and the subcutaneous tissue bluntly dissected. Keeping the neurovascular structures protected, the deep tissue was completely exposed. The pseudotumoral lesion was visualized as granular, opaque and whitish, located within the second intermetatarsal space, with no involvement of the adjacent soft tissue. The lesion was excised and removed completely, measured as 2.5 x 1.6 x 1.0 cm, and sent to pathology.

Next, attention was made toward decompression of the intermetatarsal space. The intermetatarsal ligament was identified and resected while protecting the nerve underneath. The patient tolerated the procedure well without complications, and was discharged the same day with postoperative pain management medication. Postoperative instructions were to partially bear weight with a wedged shoe for two weeks, then progressively increase weight bearing as tolerated, and return to normal activities and normal shoe wear after six weeks. The patient's postoperative follow-ups were in two weeks for wound check and suture removal in six weeks and three months.

**Figure 3:**
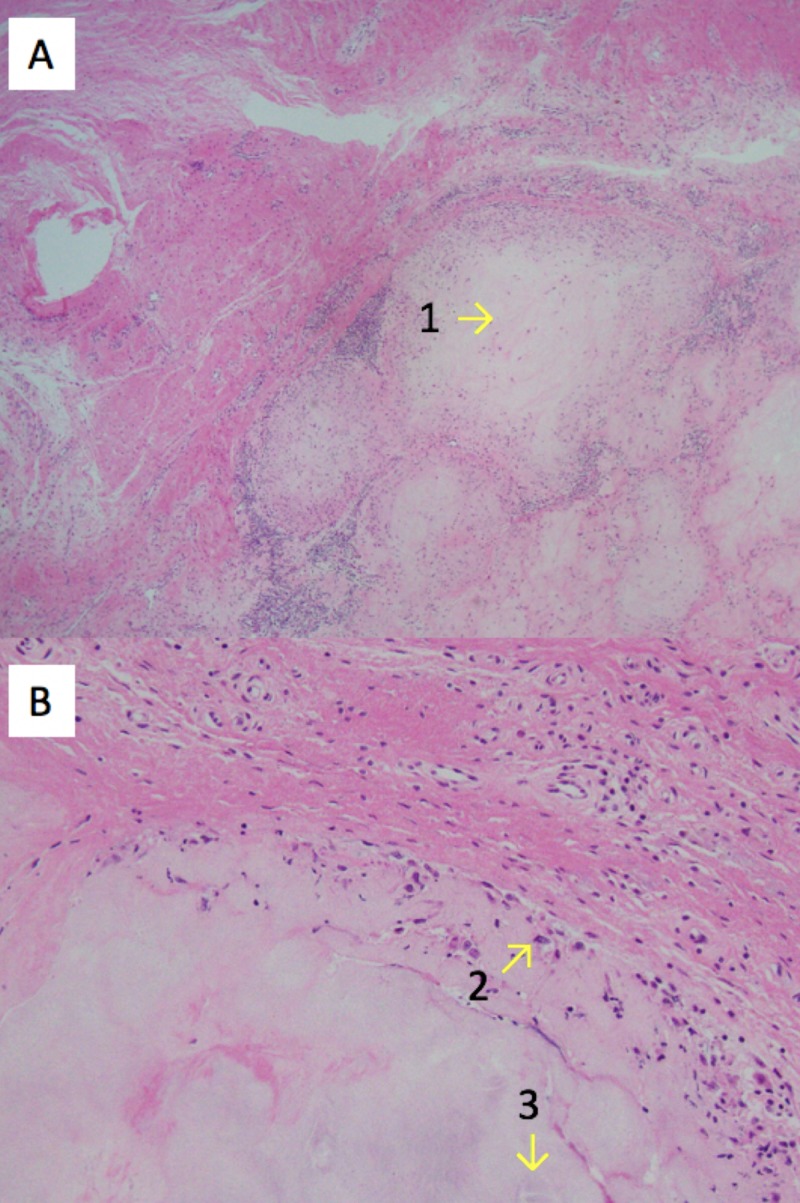
Histologic examination of the excised lesion There are well-circumscribed nodules of amorphous material (A1, 40X magnification), rimmed by histiocytes (B2, 200X magnification) and multinucleated giant cells, with needle-shaped clefts (B3) representing where the gout crystals were removed during processing.

The surgical pathology report confirmed the lesion was a gouty tophus. The histologic findings are described in Figure 3. The patient was referred to a rheumatologist for continued treatment of gout at his first postoperative follow up visit.

## Discussion

Gout is a metabolic disease, characterized by the deposition of monosodium urate crystals within joints and soft tissues. Typically, patients with this condition have a serum uric acid concentration greater than 7 mg/dL, due to an increase in production or a decrease in excretion of uric acid [5]. Gout is more commonly found in men than in women [6]. Specifically, there are nine affected males per each affected female. The presentation usually occurs during the 5th and 6th decades of life [7]. The disease naturally progresses through four phases: asymptomatic hyperuricemia, gouty arthritis, intercritical gout, and chronic tophaceous gout. Asymptomatic hyperuricemia does not ordinarily require treatment, but efforts should be made to lower urate levels through diet and lifestyle modification. Gouty arthritis typically becomes clinically evident when tissues have been exposed to hyperuricemic fluids for years. The initial clinical presentation often involves the first metatarsophalangeal joint, and subsequent flares are more likely to involve the ankles, knees, wrists, shoulder, or interphalangeal joints of the hand. Intercritical gout is the asymptomatic phase between acute gouty arthritis flares.

The cardinal sign of advanced gout is the appearance of gouty tophi. Tophi consist of a mass of monosodium urate crystals, surrounded by cells that typically characterize an inflammatory response, including immature fibroblasts, lymphocytes, plasma cells, macrophages, and foreign body giant cells [8]. They are most commonly found in the olecranon bursa, the infrapatellar and Achilles tendons, the subcutaneous tissue of the extensor surfaces of forearms, on the joints, and occasionally in the helix handset. The intradermal tophi are less frequently located on the palms and fingertips [9]. In addition, there have been reports of unusual sites or presentations of tophaceous disease. These include atypical musculoskeletal presentations causing spinal cord or nerve root compression [10-11], or involving the tarsal tunnel [12], patellar tendon [13], second metacarpal [14], or os trigonum [15]. Additionally, tophi have been reported outside of the musculoskeletal system, in the bronchus [16], mitral valve [17], liver [18], and breast [19].

Based on our literature review, there have been no reported cases of a gouty tophus located in the intermetatarsal space, or causing intermetatarsal pain or neuritis, as in our patient. These findings are typically seen in Morton’s neuroma, a characteristic lesion of the second intermetatarsal space. For this reason, Morton’s neuroma seemed the most likely diagnosis based on the patient’s history and physical exam, despite his known diagnosis of tophaceous gout. However, other differential diagnoses included a gouty tophus, a ganglionic, synovial or epidermoid cyst, intermetatarsal bursitis, granuloma annulare, infection, or a neoplasm. Thus, we ordered imaging with X-ray and MRI to further characterize the lesion. When the imaging results suggested crystal-induced arthropathy, a gouty tophus seemed the most likely diagnosis.

Most cases of tophaceous gout can be adequately treated with urate-lowering therapy. However, some tophi do not resolve with medical treatment, and surgery may be indicated based on symptom severity, compression of nearby structures, and functional impairment [20]. In our case, the patient’s gouty tophus had persisted for several years, despite treatment with allopurinol. The decision was made to proceed with surgery based upon diagnostic uncertainty, as well as the patient’s persistent pain and impaired quality of life,

This case illustrates that while Morton’s neuroma is the most common cause of a second intermetatarsal space lesion causing neuropathic pain and paresthesia, other pseudotumoral lesions such as a gouty tophus, may present similarly, and should be considered in the differential diagnosis. Thus, in the case of a space-occupying lesion, a thorough history, physical exam, and imaging should be performed, and a broad differential diagnosis should be considered. While gouty tophi can usually be resolved with medical treatment, persistent symptomatic tophi may require surgery.

## Conclusions

While gout most commonly affects the joints, it can potentially involve any area of the body. Thus, the diagnosis of a gouty tophus should be considered for a space-occupying lesion in a patient with risk factors for gout, or a known history of this disease. This is the first case reported of a gouty tophus of the second intermetatarsal interspace, presenting similar to a Morton’s neuroma. MRI helped to narrow the differential, and excisional biopsy with histologic examination definitively diagnosed the gouty tophus. As in our patient, some gouty tophi do not resolve with urate-lowering medication and may necessitate surgery.
